# Step Timing Change over Time During Wearable Exoskeleton-Assisted Gait Training: A Cross-Sectional Study

**DOI:** 10.3390/biomimetics10120820

**Published:** 2025-12-07

**Authors:** Tomohito Ito, Soichiro Koyama, Koki Tan, Shigeo Tanabe

**Affiliations:** 1 Department of Rehabilitation, Fujita Health University Hospital, Toyoake 470-1192, Aichi, Japan; tomohito.ito@fujita-hu.ac.jp; 2 Graduate School of Health Sciences, Fujita Health University, Toyoake 470-1192, Aichi, Japan; koyamas@fujita-hu.ac.jp; 3 Faculty of Rehabilitation, School of Health Sciences, Fujita Health University, Toyoake 470-1192, Aichi, Japan; 4 Department of Rehabilitation Medicine, School of Medicine, Fujita Health University, Toyoake 470-1192, Aichi, Japan; koki.tan@fujita-hu.ac.jp

**Keywords:** spinal cord injuries, exoskeleton device, rehabilitation robotics, gait disorders, neurologic, gait training

## Abstract

This study aimed to investigate the timing of foot-off and initial contact at the end of the first walking training session with a Wearable Power-Assist Locomotor (WPAL) in novice healthy users. Eight healthy volunteers with no walking experience with the WPAL participated in this study. The participants walked back and forth on a straight 5 m path for 60 min with the WPAL. We calculated the differences between the participant’s foot-off and initial contact timing, as well as the start and end timing of the pre-programmed WPAL lower-limb swing time. Data were divided into four segments of 100 data points. We calculated the median of the last 100 data points and examined whether it falls within an appropriate time range. The foot-off timing tended to be within the appropriate time range (median, −0.44 s); however, the initial contact timing was earlier than the appropriate time range (median, −0.17 s). Although some participants performed foot-off within the appropriate time range, all performed initial contact earlier than the appropriate time range. These findings may contribute to establishing practice protocols for stable walking with wearable robotic exoskeletons in patients with spinal cord injury.

## 1. Introduction

Spinal cord injury (SCI) is often caused by trauma, especially after motor vehicle collisions and sports accidents, with an incidence of 18 million new cases worldwide each year [1]. A 2018 survey reported that approximately 4600 SCIs occur annually in Japan [2].

Furthermore, a 2018 nationwide survey on the incidence and characteristics of traumatic SCI in Japan reported that patients with complete sensory and motor SCI are unable to walk independently, even after rehabilitation [3]. Patients often rely on wheelchairs as mobility devices in activities of daily living. However, long-term inactivity due to wheelchair use can result in various medical problems (e.g., joint contractures [4] and pressure sores [5], osteoporosis [6], and psychosocial problems [7]).

Wearable robotic exoskeletons (WREs) are powered exoskeletal devices designed to restore independent walking in patients with sensory- and motor-complete SCI [8,9,10,11]. Clinical trials have demonstrated that patients with complete sensory and motor SCI who used WREs were able to walk continuously for more than 60 min and over 1500 m without assistance [12,13].

In the control design of lower-limb exoskeletons, the biomimetic concept of mimicking natural human gait mechanics is considered essential [14]. In particular, gait events such as foot-off timing and initial contact timing play key roles in achieving assistance that closely approximates natural human walking [15].

To achieve independent walking with the WREs, comprehensive gait training is required [16]. However, patients with complete sensory and motor SCI experience significant challenges due to the absence of somatosensory feedback from the lower limbs below the neurological level of injury. The lack of feedback from the lower limbs, especially the sole of the foot, impedes the accurate timing of foot-off and initial contact during WREs-assisted walking. Thus, effective gait-training protocols are essential for improving gait proficiency in patients with complete sensory and motor SCI.

**Figure 1:**
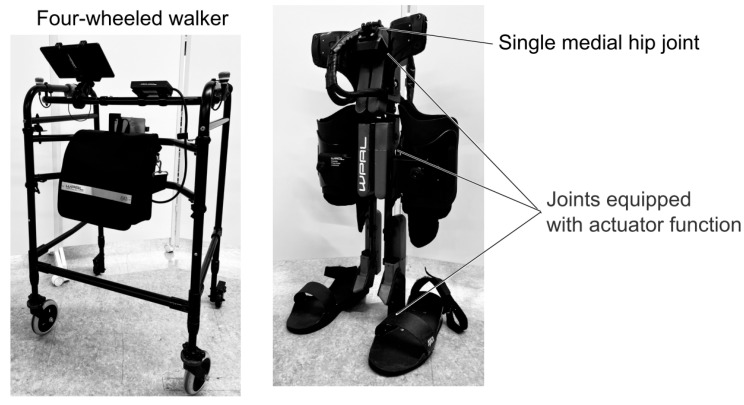
Wearable Power-Assist Locomotor.

The Wearable Power-Assist Locomotor (WPAL) is one of the WREs used to reconstruct walking in patients with SCI [17,18] (Figure 1). The WPAL is equipped with six motors across the hip, knee, and ankle joints, and each motor can generate a peak torque of 30 Nm. Before walking with the WPAL, patients set the swing time and double support time, which correspond to the pre-programmed WPAL gait temporal cycle. Patients with complete sensory and motor SCI need to coordinate upper-body movements in anterior and lateral directions, synchronized with the pre-programmed WPAL gait cycle [17,18]. Patients must perform weight shifts within the appropriate time range. The appropriate time range was defined as the phase during the double-support period when both lower-limb motions of the WPAL are stopped.

Discrepancies between the patient’s lateral weight shifts and the pre-programmed WPAL gait cycle result in errors, where the timing of foot-off and initial contact occurs outside the appropriate time range for walking with the WPAL. If the timing of foot-off and initial contact is performed outside the appropriate time range, the patient may experience falls. This is often common in the early stages of the WPAL training [19]. However, the timing of foot-off and initial contact during the early stages of the WPAL training remains poorly understood.

This study aimed to investigate the timing of foot-off and initial contact at the end of the first walking training session with the WPAL in novice healthy users.

## 2. Materials and Methods

### 2.1. Study Design

This study had a cross-sectional design. This study was conducted in accordance with the Declaration of Helsinki, and this study protocol was approved by Fujita Health University (approval no. HM22-324). This study was conducted and reported in accordance with the Strengthening the Reporting of Observational Studies in Epidemiology (STROBE) statement for observational studies [20].

### 2.2. Participants

Although the main users of the WPAL are patients with lower thoracic injuries, this study was conducted in healthy individuals to eliminate potential effects of neurological level and motor or sensory function (e.g., ASIA and Frankel Classification) on the results [21]. Using convenience sampling, eight healthy volunteers (four females, aged 21.8 ± 0.8 years) participated in this study. The participants were students at Fujita Health University. The inclusion criteria were as follows: (1) no experience of walking with the WPAL; (2) height of 155–180 cm; (3) body weight of <80 kg; (4) no recent fracture; and (5) no neuromuscular disorders that affect walking. None of the participants had previously walked with the WPAL. All participants were provided with an explanation of the study objectives and procedures before obtaining their consent. Written informed consent was obtained from all participants.

### 2.3. Experimental Setup

The experimental setup consisted of the WPAL (WPAL; ASKA Corp., Kariya, Japan), a specialized four-wheeled walker (4WW), and two tape switches (121-BP; Tape Switch Japan Corp., Matsudo, Japan) affixed to the sole of the WPAL.

A data acquisition system consisting of a computer equipped with LabVIEW 2019 (National Instruments) and an A/D converter (USB-6343; National Instruments, Austin, TX, USA) was used to record the timing of participants’ foot-off and initial contact and the pre-programmed WPAL gait cycle (i.e., swing and stance phases) simultaneously at a sampling frequency of 1 kHz. Based on a previous study, the lengths of both lower limbs of the WAPL and the heights of the 4WW were adjusted according to the body sizes of the participants [21]. The WPAL automatically moves the lower limbs in an alternating pattern according to the pre-programmed WPAL gait cycle. The pre-programmed WPAL gait cycle is defined by the swing time (i.e., alternating motions in both the WPAL lower limbs), double support time (i.e., stop time in both the WPAL lower limbs), and stride length. The WPAL gait cycle was programmed as follows: swing time 0.8 s; double support time 0.4 s; and stride length 560 mm in this study. This setup was defined based on data from a previous study [21]. Level ground, approximately 5 m long in a straight line, was used as the gait training course.

### 2.4. Experimental Procedure

Prior to walking, the researchers explained to the participants how to use the WPAL. They first described the location and operation of the Start and Stop buttons. Participants were then instructed on proper posture and on performing lateral weight shifts with voluntary upper-limb and trunk movements during walking. Finally, they were shown a video demonstrating a person who was proficient in walking with the WPAL. The walkway was a 5 m straight course, and they walked back and forth on the walkway with the WPAL for 60 min. The 60 min training time was determined based on a previous study [22]. They were closely supervised by the physical therapist (PT) to ensure safety during walking practice. The number of falls was recorded when the patient fell while walking with the WPAL. If they were unable to recover from a fall on their own, the PT assisted with their recovery and stopped the WPAL motion. One trial was defined as either walking 5 m or stopping because of a risk of falling. The steps did not include turns during walking practice. There was no feedback from the researcher regarding the timing of foot-off or initial contact while walking with the WPAL.

### 2.5. Defining the Relative Timing of Foot-Off and Initial Contact

**Figure 2:**
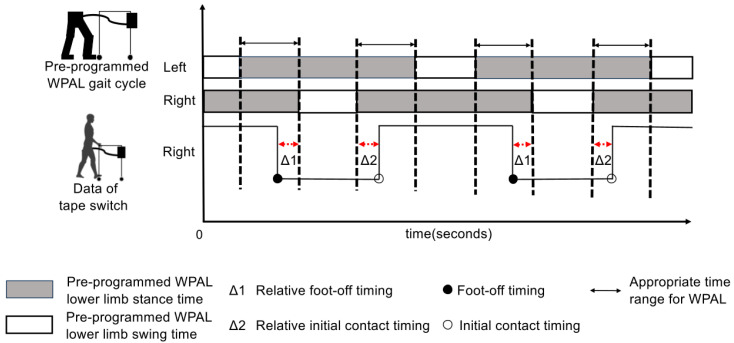
The pre-programmed Wearable Power-Assist Locomotor (WPAL) gait cycle and the data obtained from the tape switches of the right foot. The pre-programmed WPAL gait cycle is shown as a bar graph with white and gray stripes. The gray bar shows the pre-programmed stance time, whereas the white bar shows the pre-programmed swing time. The black waveform shows the data obtained from the tape switch on the right foot. These waveforms fall at foot-off and rise at initial contact. The Δ1 shows the relative foot-off timing. The Δ2 shows the relative initial contact timing. Δ1 and Δ2 are measured for each step, resulting in a total of 500 data points. The range between the dotted lines shows the double support time of the lower limb of the WPAL, which is defined as the appropriate time range for the WPAL. The black waveform rose and fell within the range enclosed by the dotted lines, indicating that foot-off and initial contact were successfully achieved. The data obtained from the tape switches on the left foot were similar.

Figure 2 illustrates the pre-programmed WPAL gait cycle and the data from the tape switch attached to the sole.

The relative foot-off timing was defined as the difference between the participant’s foot-off timing and the start timing of the pre-programmed swing time (see Figure 2). This value, between 0 and −0.4 s, indicates that the participant performed the foot-off within the appropriate time range for the WPAL. A positive value indicated that the participant performed foot-off while moving the lower limbs of the WPAL. This indicates that the timing of the foot-off was later than the appropriate time range. Values below −0.4 s indicated that the participant performed a foot-off while the lower limb of the WPAL was in motion. This indicated that the foot-off timing was earlier than the appropriate time range, indicating that the previous gait cycle had not yet been completed.

The relative initial contact timing was defined as the difference between the participant’s initial contact timing and the end timing of the pre-programmed swing time (see Figure 2). This value between 0 and 0.4 s indicates that the participant performed initial contact within the appropriate time range for the WPAL. A negative value indicates that the participant performed initial contact while moving the lower limb of the WPAL. This indicated that the initial contact timing was earlier than the appropriate time range. Values above 0.4 s indicate that the participant performed initial contact after the lower limb of the WPAL had begun moving again. This indicated that the initial contact timing was too late and the next gait cycle of the WPAL had already started.

The pre-programmed WPAL gait cycle is shown as a bar graph with white and gray stripes. The gray bar shows the pre-programmed stance time, whereas the white bar shows the pre-programmed swing time. The black waveform shows the data obtained from the tape switch on the right foot. These waveforms fall at foot-off and rise at initial contact. The Δ1 shows the relative foot-off timing. The Δ2 shows the relative initial contact timing. Δ1 and Δ2 are measured for each step, resulting in a total of 500 data points. The range between the dotted lines shows the double support time of the lower limb of the WPAL, which is defined as the appropriate time range for the WPAL. The black waveform rose and fell within the range enclosed by the dotted lines, indicating that foot-off and initial contact were successfully achieved. The data obtained from the tape switches on the left foot were similar.

### 2.6. Data Collection and Statistical Analysis

The following data were collected: (1) timing of the participant’s foot-off, (2) the participant’s initial contact timing, (3) start timing of the pre-programmed swing time, and (4) end timing of the pre-programmed swing time. We excluded the first two steps and the last two steps at both ends of each trial from the collected steps data. From this data, the steps from 0 to 400 were used for analysis. We used the analysis data to calculate the relative foot-off timing and the relative initial contact timing.

These relative foot-off and initial contact timing data were divided into four segments of 100 data points each in chronological order, and the median and quartile deviations were calculated. The segments were defined as follows: 0–100 data points as S0, 100–200 data points as S1, 200–300 data points as S2, and 300–400 data points as S3, respectively. S3 was used as the main outcome for evaluating the walking performance, specifically relative foot-off timing and relative initial contact timing, with the WPAL.

Statistical analysis was conducted to examine differences in the relative foot-off and initial contact timings between S0, S1, S2, and S3. Friedman’s test was used for comparisons. If a significant difference was observed, the Wilcoxon signed-rank test with Bonferroni correction was used. Statistical analyses were performed using the SPSS Statistics for Macintosh (version 23; IBM Corp., Armonk, NY, USA). The statistical significance level was set at 0.05.

## 3. Results

### 3.1. Summary of the Experiments

There were no falls in this study. All participants achieved independent walking. On average, all participants performed 21.8 ± 3.9 trials and took 517.3 ± 7.2 steps. Thus, the average number of analyzed steps was 435 ± 19.

### 3.2. Relative Foot-Off and Initial Contact Timing

**Figure 3:**
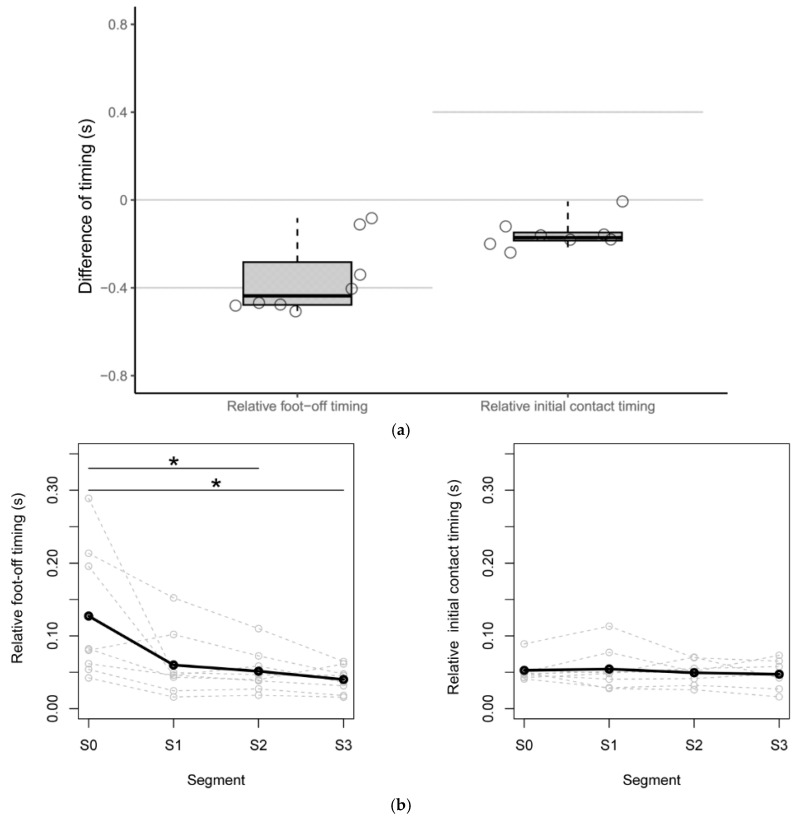
The median S3 of all participants and the comparison of the quartile deviation at each segment. (**a**) The relative foot-off timing and relative initial contact timing are shown as a boxplot. The central lines of the boxplot represent the medians, and the box limits encompass the interquartile range, which spans from the 25th to the 75th percentile. The value for each participant is shown as a point. Each gray line indicates that the motion was performed within the appropriate time range for the Wearable Power-Assist Locomotor (WPAL). (**b**) The quartile deviations for the relative foot-off timing and the relative initial contact timing. The median of the quartile deviations of the relative foot-off timing and relative initial contact timing for the eight participants are shown as black lines. Asterisks indicate statistically significant differences (*p* < 0.05).

The results of the relative foot-off and initial contact timing are shown in Figure 3a. For the foot-off timing, the median for all participants was −0.44 s (interquartile range: −0.48 to −0.28 s); three participants performed the foot-off within the appropriate time range of −0.4 to 0 s. For the initial contact timing, the median for all participants was −0.17 s (−0.19 to −0.15 s); all participants performed initial contact earlier than the appropriate time range.

### 3.3. Change over Time in Quartile Deviations of the Foot-Off and Initial Contact Timing

The results of the quartile deviations of the relative foot-off timing and relative initial contact timing for the eight participants are shown in Figure 3b. In the comparison of the quartile deviation for the relative foot-off timing at each segment, there was a significant decrease between S0 and S2 and between S0 and S3 (*p* = 0.047, *p* = 0.047). For the relative initial contact timing, the values were around 0.05 s from S0 to S3. No significant differences were observed in comparisons across segments.

## 4. Discussion

This study was conducted to investigate the timing of foot-off and initial contact at the end of the first walking training session with the WPAL in novice healthy users. Three of the eight participants exhibited foot-off times within the appropriate time range. In contrast, the initial contact timing was earlier than the appropriate time range for all participants. The variability of the relative foot-off timing decreased by 100 steps, whereas the variability of the relative initial contact timing remained unchanged at less than 0.05 s.

The results of this study revealed differences between the participant’s foot-off timing and the start timing of the pre-programmed swing time, and between the participant’s initial contact timing and the end timing of the pre-programmed swing time after achieving independent walking with the WPAL in novice healthy users who had never walked with the WPAL and practiced without external feedback. This was especially evident in the timing of initial contact. This study contributes to the establishment of walking practice protocols with other WREs.

Three of the eight participants performed foot-off within the appropriate time range for the WPAL. The remaining five participants performed foot-off earlier than the appropriate time range for the WPAL. Although this study did not explain the reason for this result, individual variations in motor learning might influence these differences. The acquisition of the new motor task is related to the mechanisms of motor learning. Several studies have shown inter-individual differences in learning new motor tasks [23,24]. Factors related to motor learning include trainability, talent [25,26], and the ability to adopt and optimize motor skills [27,28]. We believe that the differences in the ability to adopt and optimize motor skills exist because the participants in this study had different exercise habits and histories. A previous study that used golf as a task considered exercise history [29].

Regarding the initial contact timing, all participants moved approximately 0.2 s earlier than the pre-programmed double support time. The reason for this result is unclear from our data; however, the difference between the swing time of walking without the WPAL and the pre-programmed swing time may have been a contributing factor. The swing time while walking without the WPAL is approximately 0.4 s [30]; however, the pre-programmed swing time is 0.8 s. We believe that the difference in walking rhythm between walking with and without the WPAL led to this result.

Based on previous studies [31,32], this study examined proficiency through performance variability to confirm differences in relative foot-off and initial contact timing. The results of the quartile deviation of the relative foot-off timing showed that while there was variability of more than 0.1 s at S0, it gradually decreased to less than 0.1 s by S3. We consider variability within 0.1 s to be small during walking because the difference in swing time among healthy adults during normal walking is greater than 0.1 s [33]. Exercise repetition is important for motor learning [34]. These results suggest that repeated walking practice with the WPAL is effective in improving the foot-off timing.

The results of the quartile deviation of the relative initial contact timing showed that the variability in the relative initial contact timing among all participants from S0 to S3 was less than 0.1 s. We consider this result to be the reason why there were no significant differences compared to S0 in any segment.

All participants performed initial contact earlier than the appropriate time range at S3. Additionally, no significant differences were observed in the relative timing of initial contact across each segment. Therefore, we believe that motor learning did not alter initial contact performance during walking with the WPAL. Several studies have indicated that providing feedback on exercise errors can improve skill levels and assist in achieving target tasks [35,36,37]. External feedback is more effective than internal feedback for motor learning [29,38]. The results of this study suggest that external feedback might be important for the initial contact timing during walking practice with the WPAL.

This study examined the timing of foot-off and initial contact during weight shifting in novice healthy users. Walking with ReWalk (ReWalk Robotics, Inc., Marlborough, MA) is performed based on the angle of the torso and the movement of the center of gravity [39]. Ekso (Ekso Bionics; Richmond, CA, USA) has two gait training modes: one is automatically controlled by the caregiver using a button, and the other is initiated by the operator’s own weight shift [40]. All patients with SCI need to learn to time lateral weight shifts during WRE movements to maintain a stable standing posture [41]. Two studies have been conducted on the establishment of practice protocols for WREs [42,43]. This study contributes to the establishment of a practical protocol for WREs in patients with SCI.

This study has some limitations. This study was conducted on healthy volunteers under the same conditions as patients with lower thoracic injuries. The WPAL automatically moves the lower limbs according to the pre-programmed WPAL gait cycle and is not influenced by the user’s voluntary movements. Therefore, by suppressing voluntary lower-limb muscle activity, the condition of lower-thoracic SCI patients can be replicated, suggesting that the experimental results in individuals with SCI would be similar to those in this study. However, caution should be exercised when comparing healthy volunteers and individuals with SCI. Additionally, in this study, the gait cycle of the WPAL was standardized based on previous research; however, in actual clinical settings, the gait cycle may vary among patients. Because such variations could influence the timing of initial contact and foot-off, future work should examine the relationship between the timing of initial contact and foot-off and the gait cycle of the WPAL.

Patients with SCI often experience sensory deficits and spasticity in the lower limbs [44,45], which can affect the timing of foot-off and initial contact during walking with the WPAL. Future studies should investigate walking with the WPAL in patients with various levels of SCI to clarify the differences in outcomes resulting from physical function. Furthermore, this study was performed without external feedback to prevent facilitation of motor learning during walking with the WPAL. Subsequent investigations should examine the impact of external feedback on the timing of foot-off and initial contact during walking with the WPAL.

## 5. Conclusions

This study investigated the foot-off and initial contact timing at the end of the first WPAL walking training session in novice healthy users. Each participant walked for approximately 60 min without external feedback, during which the foot-off and initial contact timing were recorded. We then calculated the differences between each participant’s foot-off and initial contact timing. As the result, the foot-off was performed within the appropriate time range. Conversely, the initial contact was performed earlier than the appropriate time range for the WPAL. These findings underscore the importance of external feedback on the timing of initial contact during the practice phase.

## Data Availability

The data supporting the findings of this study are available from the corresponding author upon reasonable request.

## Abbreviations

The following abbreviations are used in this manuscript:

WAPL	Wearable Power-Assist Locomotor
SCI	Spinal cord injury
WREs	Wearable robotic exoskeletons
PT	Physical therapist
